# Outcome of patients with local recurrent gynecologic malignancies after resection combined with intraoperative electron radiation therapy (IOERT)

**DOI:** 10.1186/s13014-016-0622-x

**Published:** 2016-03-18

**Authors:** Nathalie Arians, Robert Foerster, Joachim Rom, Matthias Uhl, Falk Roeder, Jürgen Debus, Katja Lindel

**Affiliations:** National Center for Radiation Oncology (NCRO), Heidelberg Institute for Radiation Oncology (HIRO), Heidelberg, Germany; Department of Radiation Oncology, University Hospital Heidelberg, Heidelberg, Germany; Department of Obstetrics and Gynecology, University of Heidelberg, Heidelberg, Germany; Department of Radiation Oncology, University Hospital Munich (LMU), Munich, Germany; CCU Molecular Radiation Oncology, German Cancer Research Center (DKFZ), Heidelberg, Germany

**Keywords:** IOERT, Pelvic exenteration, Recurrent gynecologic cancer, Cervical cancer, Endometrial cancer, Vulvar cancer

## Abstract

**Background:**

Treatment of recurrent gynecologic cancer is a challenging issue. Aim of the study was to investigate clinical features and outcomes of patients with recurrent gynecologic malignancies who underwent resection including IOERT (intraoperative electron radiation therapy) with regard to clinical outcome and potential predictive factors or subgroups that benefit most from this radical treatment regime.

**Methods:**

A total of 36 patients with recurrent gynecologic malignancies (cervical (*n* = 18), endometrial (*n* = 12) or vulvar cancer (*n* = 6)) were retrospectively identified through hospital databases in accordance with institutional ethical policies.

Patient characteristics and outcomes were assessed. Survival data was analyzed using the Kaplan-Meier-method and log-rank-test, categorical variables were analyzed with chi-square-method.

**Results:**

For the entire cohort 1-/2-/5-year Overall Survival (OS) was 65.3 %/36.2 %/21.7 %. Patients with endometrial, cervical, and vulvar carcinoma had a 1-/2-/5-year OS of 83.3 %/62.5 %/50 %, 44.5 %/25.4 %/6.4 %, and 83.3 %/16.7 %/16.7 %, respectively. Patients with endometrial carcinoma showed a significantly better OS (*p* = 0.038).

1-/2-/5-year Local Progression-free Survival (LPFS) for the entire cohort was 44.1 %/28 %/21 % with 76.2 %/61 %/40.6 % for endometrial, 17.2 %/0 %/0 % for cervical, and 40 %/20 %/20 % for vulvar cancer, respectively. Patients with endometrial cancer showed a significantly (*p* = 0.017) and older patients a trend (*p* = 0.059) for a better LPFS.

1-/2-/5-year Distant Progression-free Survival (DPFS) for the entire cohort was 53.1 %/46.5 %/38.7 % with 74.1 %/74.1 %/74.1 % for endometrial, 36.7 %/36.7 %/0 % for cervical, and 60 %/30 %/30 % for vulvar cancer, respectively. There was a significantly better DPFS for older patients (*p* = 0.015) and a trend for a better DPFS for patients with endometrial carcinoma (*p* = 0.075).

**Conclusion:**

The radical procedure of resection combined with IOERT seems to be a valid curative treatment option for patients with recurrent endometrial carcinoma with 5-year survival rates of 50 %. For patients with cervical or vulvar cancer this treatment should be considered a rather palliative one and must be weighted carefully against other treatment options like chemotherapy, targeted therapies or new highly conformal radiotherapy techniques.

**Electronic supplementary material:**

The online version of this article (doi:10.1186/s13014-016-0622-x) contains supplementary material, which is available to authorized users.

## Background

Every year almost 95,000 women are diagnosed with a gynecologic malignancy [1]. Fortunately, the majority can be cured. Around 30 % will experience cancer recurrence, often with a dismal prognosis. Endometrial carcinoma represents the most common gynecologic malignancy. Patients with endometrial carcinoma generally have a good prognosis as most women are diagnosed in an early stage and can by cured by surgery. 5-year survival rates of 80–90 % have been reported [2–4]. Risk of local recurrence – defined as isolated vaginal recurrence - can even be lowered by the additional use of adjuvant pelvic radiotherapy as shown in the PORTEC-trial (incidence of vaginal recurrence of 2 % in the radiotherapy group versus 8 % in the control group) [4–7]. Nevertheless, the prognosis for women with advanced stage disease or high-risk histologies is poor with 5-year survival rates of 57 % for Stage III and 19 % for Stage IV tumors [4, 8]. All in all, 15 % of all women with endometrial cancer experience recurrence, with more than 50 % occurring within 2 years of primary treatment [4].

The incidence of cervical carcinoma has decreased in the Western countries over the last years but still remains a significant public health problem worldwide. The risk of recurrence after primary treatment depends on the tumor stage as well as on other risk factors like tumor size, lymphonodal status, deep stromal invasion or parametrial and surgical margin involvement [9, 10]. Most recurrences of cervical carcinoma occur within 18–24 months from the time of diagnosis [11, 12], whereas 70 % of the recurrences are pelvic ones. The 5-year survival rate after recurrence in high-risk patients is less than 10 % [13]. The site of relapse is a significant prognostic factor [13] with worse results for patients with pelvic wall recurrences [12, 14–17].

Vulvar cancer represents 5 % of all gynecologic malignancies [8, 18]. Inguinal and femoral lymphatic node involvement is the most important prognostic factor for survival with a 5-year survival rate of 70–93 % for patients without lymph node involvement and only 25–41 % for those with positive lymph nodes [19, 20]. Local or distant recurrences are generally difficult to treat and the 5-year survival rate is less than 5 % [21].

In general, defining the treatment of choice for recurrent gynecologic cancer is a challenging issue. Many different factors must be taken into consideration such as the type of primary therapy, the site of recurrence, the disease-free interval, the patient’s symptoms and performance status and the degree to which any given treatment might be beneficial [12].

Pelvic exenteration may be considered in cases of recurrent or advanced-stage tumors with bladder and/or rectum infiltration neither extended to the pelvic side walls nor showing any distant metastases. High overall morbidity rates of about 70 % are still reported [12, 22, 23]. The postoperative mortality has decreased over time with nowadays 0–5.3 % [24, 25]. However, 5-year survival rates of 20–73 % after pelvic exenteration have been reported depending on disease situation and stage [12].

If resection or pelvic exenteration alone is not likely to produce satisfactory results, IOERT should be taken into consideration to increase the chance of local control. Improved outcome especially in case of close or positive margins has been reported for other pelvic diseases like locally recurrent rectal cancer previously [26]. The recommended doses depend on the exposure due to prior radiation treatment and the extent of residual tumor disease after resection, ranging from 10 to 20 Gy [12].

However, due to the rarity of the mentioned disease situations and the restricted availability of IOERT only scarce data on efficacy and toxicity of the combined treatment approach exists. We therefore analyzed our patients with recurrent gynecologic malignancies who underwent resection including IOERT at University Hospital Heidelberg between 2002 and 2014 with regard to clinical outcome and potential predictive factors or subgroups that benefit most from this radical treatment regime.

## Patients and methods

There were 36 patients with recurrent gynecologic malignancies including cervical (=18), endometrial (*n* = 12), and vulvar cancer (*n* = 6) between 2002 and 2014 who had resection combined with IOERT at University Hospital Heidelberg.

Computerized database was used to perform a review of medical records in order to abstract patient and treatment characteristics. All data were collected retrospectively and in accordance with institutional ethical policies. The following clinical data were collected: age, tumor type, date of first diagnosis, prior treatment, time to recurrence, pattern of recurrence including organ infiltration, lymphonodal status, existence of a lymphangiosis carcinomatosa, extent of resection, resection margins, performance of IOERT including dose, energy and cone size, onset and localization of distant metastases and further local recurrences, date and cause of death, toxicities and treatment associated complications.

There were no restrictions to the type of prior treatment (Table 1). In the cervical cancer group 17 patients had a tumor resection before. 5 patients had an EBRT (median dose 52.2 Gy, range 45–55.6 Gy), 8 patients had concurrent chemotherapy with cisplatin or cisplatin/5-fluorouracil (median dose 50.4 Gy, range 50.4–58.5 Gy), 2 patients had an additional brachytherapy as a boost (median dose 30 Gy, range 20–40 Gy) and another 2 patients had brachytherapy in a separate time frame from EBRT (15 Gy for one patient, dose information n.a. for the other patient). 4 patients didn’t have any radiotherapy before resection + IOERT. Two patients had chemotherapy alone in the course of their treatments. All patients with endometrial cancer had a hysterectomy before. 7 patients received vaginal brachytherapy (median dose 21 Gy, range 15–30 Gy). One patient had additional EBRT (50.4 Gy with 20 Gy brachytherapy as a boost, and later another EBRT with 50 Gy) and another patient had EBRT alone (50.4 Gy). Two patients had chemotherapy alone in the course of their treatments. All patients with vulvar cancer had a tumor resection, 5 had an additional EBRT (median dose 50.4 Gy, range 50–61.2 Gy), one patient had concurrent EBRT and chemotherapy with cisplatin/5-fluorouracil (dose information n.a.).

**Table 1:** Prior treatment regimens and dose specifications by tumor type

	Number	Median dose	Dose range	IOERT	IOERT
				median dose	dose range
A. Cervical cancer					
Cervical cancer (*n* = 18)					
Resection	17	–	–	–	–
EBRT alone	5	52.5	45–55.6	15	12–15
EBRT + CHT	8	50.4	50.4–58.5	15	10–18
EBRT + Brachytherapy	4	50 + 20	36–50.4 + 15–40	15	12–15
Brachytherapy alone	0	–	–	–	–
CHT alone	2	–	–	–	–
No prior radiation	4	–	–	12	10–15
B. Endometrial cancer					
Endometrial cancer (*n* = 12)					
Resection	12	–	–	–	–
EBRT alone	1	50.4	–	15	15
EBRT + CHT	0	–	–	–	–
EBRT + Brachytherapy	1	50.4 + 20; 50	–	10	10
Brachytherapy alone	7	21	15–30	15	10–15
CHT alone	2	–	–	–	–
No prior radiation	3	–	–	15	10–15
C. Vulvar cancer					
Vulvar cancer (*n* = 6)					
Resection	6	–	–	–	–
EBRT alone	5	50.4	50–61.2	15	12–15
EBRT + CHT	1	n.a.	n.a.	15	15
EBRT + Brachytherapy	0	–	–	–	–
Brachytherapy alone	0	–	–	–	–
CHT alone	0	–	–	–	–
No prior radiation	0	–	–	–	–

Patients with recurrent gynecologic cancer were generally discussed in an interdisciplinary setting. If there were no other curative options left and patients were eligible for resection and IOERT with expected reasonable morbidity and toxicity this therapeutic option was offered to the patients. The extent of resection was depending on the pattern of tumor recurrence. All patients received IOERT. The operations were performed by gynecologic, urologic, visceral or even plastic and vascular surgeons from the University Hospital Heidelberg. All recurrences were histologically confirmed. The technique of IOERT used at our institution has been described previously [26]. Briefly, IOERT was performed in a dedicated operation room with an integrated linear accelerator capable of delivering 6–18 MeV electrons. The target area was defined in correspondence with the surgeon and usually included the high risk area for positive margins with a safety margin of 1 cm. Uninvolved radiosensitive tissues (for example major nerves, ureters and bowel structures) were displaced or protected by lead shields whenever possible. The median IOERT dose was 15 Gy (range 10–18 Gy) with a median energy of 8 MeV (range 6–15 MeV), prescribed to the 90 %-isodose. IOERT dose was usually restricted to 10–12 Gy, if major nerves had to be included into the radiation field. Detailed information about the IOERT doses depending on tumor type and previous treatment regimens are listed in Table 1.

Further treatment after resection combined with IOERT was very variable, depending on the clinical characteristics of each patient. One cervical cancer patient without any previous radiation therapy received adjuvant radiochemotherapy with a dose of 45 Gy combined with cisplatin 30 mg/m^2^ body surface weekly (IOERT dose: 12 Gy). Another cervical cancer patient stopped adjuvant EBRT after 5.4 Gy. One vulvar cancer patient with prior EBRT with 56 Gy had adjuvant radiochemotherapy with 45 Gy and cisplatin 40 mg/m^2^ body surface weekly (IOERT dose: 15 Gy). Two patients with endometrial cancer had adjuvant EBRT. One patient without any previous radiation received 41.4 Gy (IOERT dose: 10 Gy). The other one already had a brachytherapy with 3 × 8 Gy and received another 40 Gy EBRT after resection and IOERT (IOERT dose: 15 Gy).

Overall Survival (OS), Local Progression-free Survival (LPFS) and Distant Progression-free Survival (DPFS) were evaluated. Statistical events were defined as death from any cause (OS), any loco-regional relapse (LPFS) and occurrence of any distant metastases or positive lavage cytology (DPFS). Time to event data was measured from the date of resection and IOERT and analyzed using the Kaplan-Meier method. Subgroups were compared with the log-rank-test, categorical variables with the chi-square-method, using SPSS version 20. A *p* value of < 0.05 was considered statistically significant.

## Results

### Patient characteristics

18 patients with recurrent cervical cancer (5 with adenocarcinoma and 13 with squamous cell carcinoma), 12 patients with recurrent endometrial, and 6 patients with recurrent vulvar cancer were identified. Patients had a median follow-up of 14 months (range: 0.1–154 months). The demographic and clinicopathological characteristics are summarized in Table 2. Median age at date of resection and IOERT was 53.5 years. The median time to recurrence from the date of first diagnosis was 55.9 months (range: 7–227 months). 20 of the 36 patients (55.6 %) had an extensive tumor recurrence with infiltration of either bladder and/or rectum, 13 patients (36.1 %) showed positive lymph nodes and 7 patients (19.4 %) a lymphangiosis carcinomatosa. The extent of resection was depending on the pattern of tumor recurrence, ranging from excision to pelvic exenteration including vessel resection. Gross complete resection had been attempted in all patients. In 15 cases R0-resection could be achieved (41.7 %), 14 patients had R1-resection (38.9 %), and only 1 patient R2-resection (2.8 %). 6 patients had complete gross but microscopically unclear resection margins (Rx).

**Table 2:** Demographic and clinicopathologic characteristics

	Number	Mean	Median	Range/Percent
Age at initial diagnosis		50.4	49	27–76
Age at time of resection + IOERT		54.6	53.5	27–83
Time to recurrence (months)		25	55.9	7–227
Primary tumor site				
Cervix	18			50 %
Endometrium	12			33.3 %
Vulva	6			16.7 %
Histology				
Squamous cell carcinoma	19			52.8 %
Adenocarcinoma	13			36.1 %
Serous	3			8.3 %
Mucinous	1			2.8 %
Organ infiltration (bladder and/or rectum)	20			55.6 %
Positive lymph nodes	13			36.1 %
Lymphangiosis carcinomatosa	7			19.4 %
Resection margins				
R0	15			41.7 %
R1	14			38.9 %
R2	1			2.8 %
Rx	6			16.7 %
Applied dose (Gray) at IOERT		13.8	15	10–18

### Survival data

Death was documented in 25 patients - 15 with cervical, 5 with endometrial, and another 5 with vulvar cancer. Median OS was 14 months (range 0.1–153.5 months; 24.5 months for endometrial, 10.3 months for cervical, 16 months for vulvar cancer) (Table 3).

**Table 3:** Survival outcomes by tumor type

	Number	Percent	Median time (months)
Loco-regional recurrence	18	50 %	6
Cervical cancer	10	55.6 %	4.7
Endometrial cancer	4	33.3 %	13.1
Vulvar cancer	4	66.7 %	7
Distant metastases	16	44 %	4.6
Cervical cancer	10	55.6 %	3.6
Endometrial cancer	3	25 %	11.3
Vulvar cancer	3	50 %	8.4
Death	25	64 %	14
Cervical cancer	15	83.3 %	10.3
Endometrial cancer	5	41.7 %	24.5
Vulvar cancer	5	83.3 %	16

**Fig. 1:**
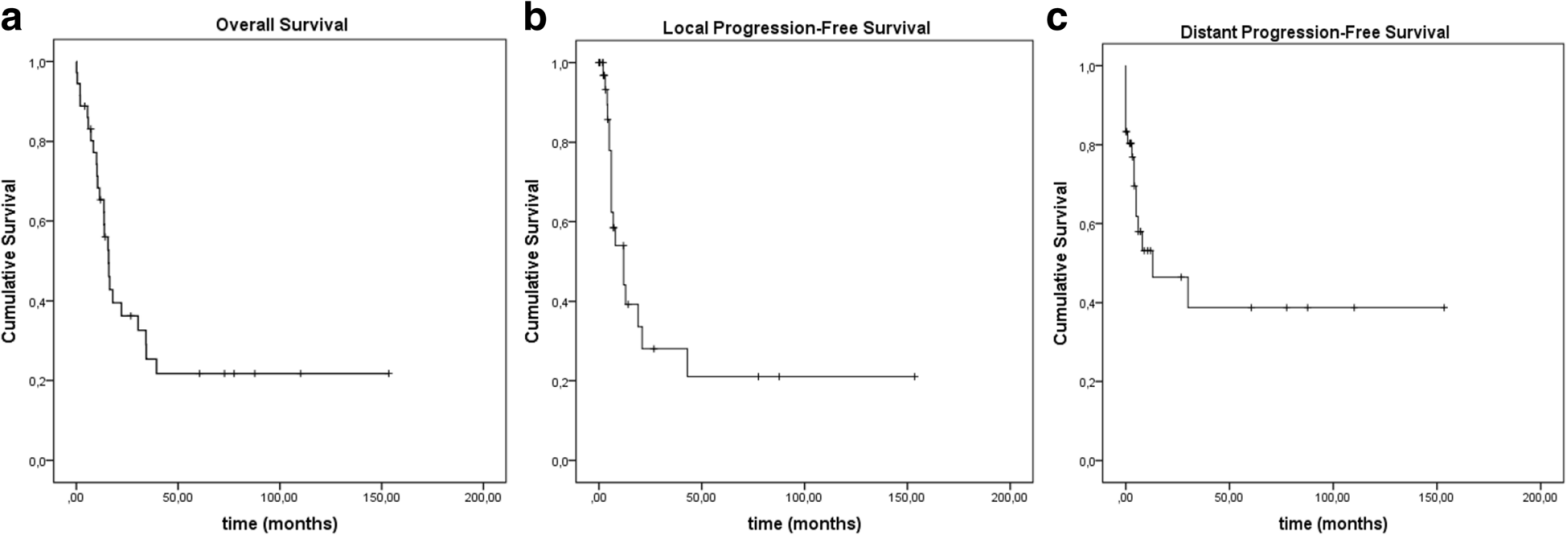
Kaplan-Meier curves of OS (**a**), LPFS (**b**) and DPFS (**c**) of the whole group

For the entire cohort 1-year, 2-year and 5-year OS was 65.3, 36.2 and 21.7 %, respectively (Fig. 1, Table 4). For patients with endometrial carcinoma 1-year, 2-year and 5-year OS was 83.3, 62.5, and 50 %. For patients with cervical cancer 1-, 2-, and 5-year OS was 44.5, 25.4, and 6.4 %, and for patients with vulvar cancer 83.3, 16.7 and 16.7 %, respectively (Table 4).

**Table 4:** Survival outcomes based on the Kaplan-Meier method

	Whole group			Endometrial cancer			Cervical cancer			Vulvar cancer		
	1 year	2 year	5 year	1 year	2 year	5 year	1 year	2 year	5 year	1 year	2 year	5 year
OS	65.3 %	36.2 %	21.7 %	83.3 %	62.5 %	50 %	44.5 %	25.4 %	6.4 %	83.3 %	16.7 %	16.7 %
LPFS	44.1 %	28 %	21 %	76.2 %	61 %	40.6 %	17.2 %	0 %	0 %	40 %	20 %	20 %
DPFS	53.1 %	46.5 %	38.7 %	74.1 %	74.1 %	74.1 %	36.7 %	36.7 %	0 %	60 %	30 %	30 %

**Fig. 2:**
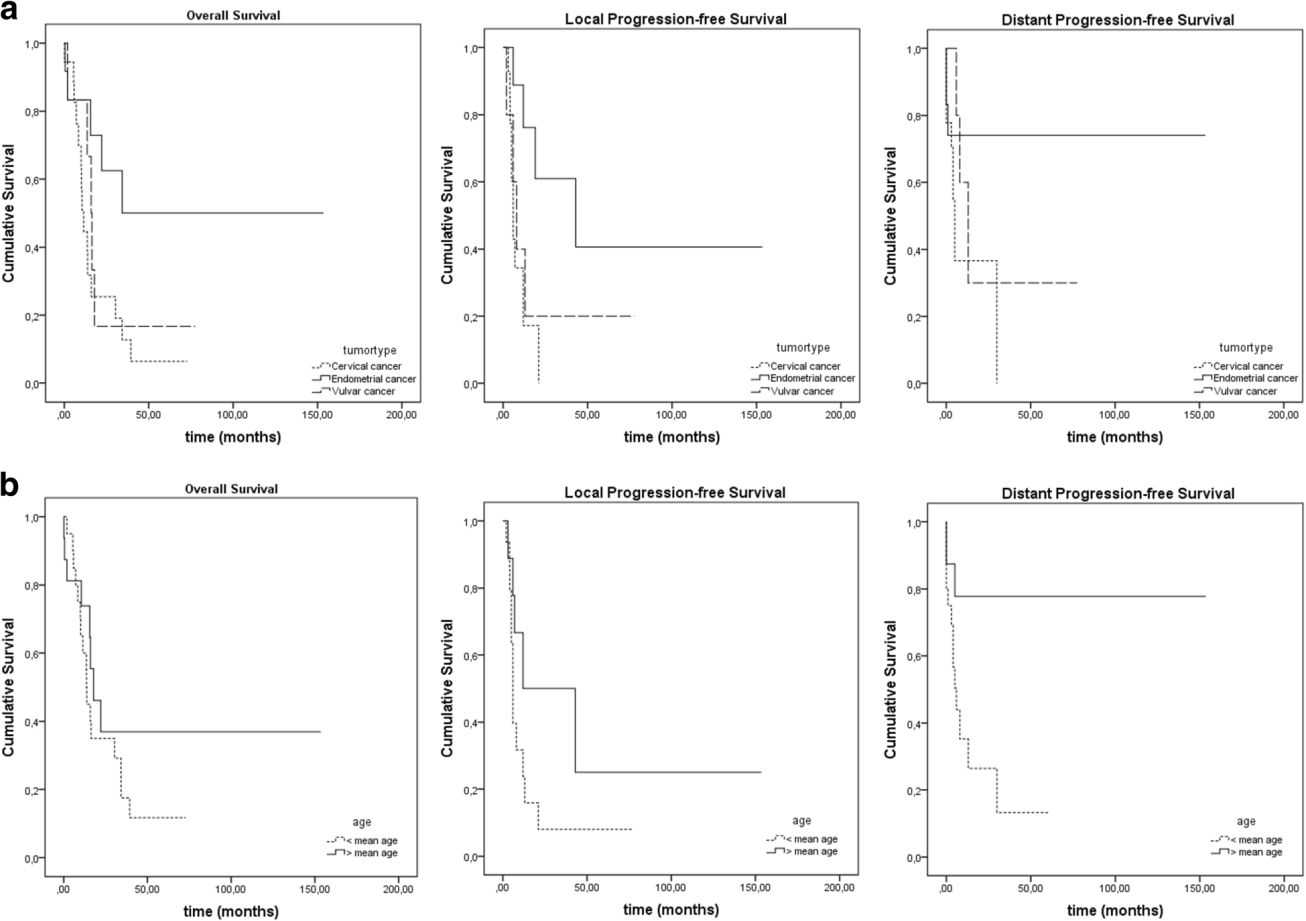
Kaplan-Meier curves of OS, LPFS and DPFS by tumortype (**a**) and age (**b**)

Patients with endometrial carcinoma showed a significant better OS (*p* = 0.038, log rank) (Fig. 2).

Local pelvic progression was documented in 18 patients - 10 with cervical, 4 with endometrial and another 4 with vulvar cancer. Median time to local progression was 6 months (range 2–153.5 months; 13.1 months for endometrial, 4.7 months for cervical, 7 months for vulvar cancer) (Table 3).

1-year LPFS was 44.1 % (76.2 % for endometrial, 17.2 % for cervical, 40 % for vulvar cancer), 28 % at 2 years (61 % for endometrial, 0 % for cervical, 20 % for vulvar cancer) and 21 % at 5 years (40.6 % for endometrial, 0 % for cervical, 20 % for vulvar cancer) (Fig. 1, Table 4). Patients with endometrial cancer showed a significant (*p* = 0.017) and older patients a trend (*p* = 0.059) for a better LPFS (Fig. 2).

16 patients developed distant metastases during follow-up - 10 with cervical, 3 with endometrial and vulvar carcinoma, respectively. Median time to distant progression was 4.6 months (range 0–153.5 months; 11.3 months for endometrial, 3.6 months for cervical and 8.4 months for vulvar cancer) (Table 3). 1-year DPFS was 53.1 % (74.1 % for endometrial, 36.7 % for cervical, 60 % for vulvar cancer), 46.5 % at 2 years (74.1 % for endometrial, 36.7 % for cervical, 30 % for vulvar cancer) and 38.7 % at 5 years (74.1 % for endometrial, 0 % for cervical, 30 % for vulvar cancer) (Fig. 1, Table 4). There was a significant better DPFS for older patients (*p* = 0.015) and a trend for a better DPFS for patients with endometrial carcinoma (*p* = 0.075) (Fig. 2). 12 of the 16 patients with distant metastases also had loco-regional recurrence. Statistical analysis showed no significance for LPFS as positive predictive factor for DPFS or the other way around.

Further statistical analysis of possible predictive factors was performed. Histology, time to recurrence, lymphonodal status, existence of lymphangiosis carcinomatosa, organ infiltration, resection margins, and IOERT dose were assessed. None of the mentioned parameters showed a statistically significant influence on OS, LPFS or DPFS (see Figure S1 in Additional file 1). Furthermore, statistical analysis using the chi square method showed that no two parameters were statistically significant dependent on each other, also not the two parameters tumor type and age.

A subgroup analysis of all patients with recurrent cervical cancer didn’t show a significant influence of tumor histology on OS (*p* = 0.348), LPFS (*p* = 0.465) or DPFS (*p* = 0.688).

### Complications/Toxicity

All in all, 50 %/61 %/67 % of patients with recurrent endometrial/cervical/vulvar cancer developed complications. 3 patients (8.3 %) died because of postoperative complications with hemorrhagic shock (1 patient with cervical, endometrial, and vulvar carcinoma, respectively). 3 patients (8.3 %) developed problems with the femoral head. IOERT dose was usually restricted to 10–12 Gy if major nerves had to be included in the radiation field. Nevertheless, nerval affections occurred in 4 patients (11.1 %) including paresis of nerves of the plexus lumbosacralis, the sciatic and femoral nerve, paresthesia, incontinence and pain. In one case sacral nerve roots were in the radiation field, so that the dose was restricted to 12 Gy (patient already received 20 Gy brachytherapy before). In another case the sciatic nerve had to be prepared to resect the tumor, which was later blocked by lead sheets during IOERT, so that a dose of 15 Gy could be administered to the left iliac axis (prior EBRT with 45 Gy). The third patient already had EBRT two times before (50 Gy and 39.6 Gy + 28 Gy brachytherapy boost) and developed a paralysis of the adductor muscles after resection + IOERT to the symphysis with 15 Gy. The fourth patient had a tumor infiltration of the whole right pelvic side wall including the iliac vessels, so that IOERT dose was restricted to 12 Gy. Postoperative healing disorders and infections occurred in 22.2 % with 5 patients suffering from necrosis or wound dehiscence and 3 patients suffering from abscesses/fluid retention. For 3 patients (8.3 %) a lymphedema or lymphocele was documented. 2 patients (5.6 %) developed a thrombosis.

## Discussion

In this study, patients with recurrent cervical carcinoma had a 1-year OS of 44.5 % with only 6.4 % alive after 5 years. Local control rates were even worse with 0 % LPFS after 2 years. Data from Qiu et al. of 121 patients with recurrent Stage I-II cervical cancer who received primary radical hysterectomy showed a 5-year survival of 22.3 % [13]. The site of relapse was a significant prognostic factor. Patients with vaginal recurrence had a significantly better 5-year overall survival with 57.3 %, whereas patients with recurrence at pelvis or distant sites had a 5-year survival of 41.8 and 10.9 %, respectively [13]. The different survival outcomes compared to the present study may be explained by the different patient collectives. In our cohort we had many patients with advanced stage cervical cancer who already had many prior therapies including radical hysterectomy, radiotherapy, radiochemotherapy or often a combination of several modalities, so that the collective was a priori a negatively selected one. Furthermore, salvage treatment in the cohort of Qiu et al. consisted of different treatment modalities including surgery, radiotherapy, chemotherapy or a combination of these, whereas our patients all received a resection combined with IOERT.

If resection or radiation of recurrent cervical cancer is not possible, palliative platin-based chemotherapy has been the only treatment option until recently. Mean Overall Survival rates ranging from 6.5 to 18.3 months have been reported with mean Progression-free Survival rates of 2.8 to 6.9 months [27–30]. The tumor response to chemotherapy depends on many factors. Black race, performance status > 0, pelvic disease, previous radiosensitizer and time interval from diagnosis to first recurrence < 1 year have been identified as risk factors for poor response [31]. In our cohort, 10 patients with cervical cancer already had prior chemotherapy treatment and all patients had pelvic disease so that a good response of the tumor to chemotherapy would have been unlikely. With 5-year survival rates of 6.4 % after resection and IOERT and even poor local control rates in our cohort, this treatment should also be considered a rather palliative one. It is a very individual decision in which case of recurrent cervical carcinoma an extensive resection combined with IOERT is reasonable and ethically acceptable.

Treatment for patients with loco-regional recurrence of endometrial cancer in a previously irradiated field is discussed controversially. Surgical resection with or without IOERT is one curative treatment option. 5-year survival rates of 40–45 % after pelvic exenteration have been reported [32, 33]. There are limited data on the use of IOERT for recurrent endometrial cancer. Chemotherapy has also been shown to have activity in endometrial cancer. Median PFS of 8.3 months and median OS of 15.3 months have been demonstrated by the GOG 177 protocol using paclitaxel, doxorubicin and cisplatin, but with a high incidence of peripheral neuropathy (12 %) [34]. In our cohort, patients with recurrent endometrial carcinoma showed a median survival of 24.4 months and a 5-year Overall Survival of 50 % with 5-year loco-regional and distant control rates of 40.6 and 74.1 %, respectively. In general, patients’ age seemed to be a prognostic parameter, as older patients had a better local and distant progression-free survival. This might be due to the fact that endometrial carcinoma is generally associated with older age. However, statistical analysis using the chi-square-method didn’t show any statistically significant dependence of tumor type and age, as the patients’ collective was too small.

A recent study suggested that in recurrent endometrial carcinoma residual disease is the most important prognostic factor after resection for both Progression-free Survival and Overall Survival [35]. Estimated 5-year PFS/OS was 42 %/60 % in optimally and 19 %/30 % in suboptimally cytoreduced patients [35]. Furthermore, literature suggests that patients with complete gross resection of gynecologic recurrences also have the greatest benefit from IOERT [1, 36, 37]. The survival outcomes in our group were similar to those of the optimally cytoreduced patients in the cohort of Papadia et al. In the present study the resection status seemed to have no influence on outcome as already shown in other studies evaluating the effect of IOERT [38]. Patients with free resection margins (R0) didn’t show a better OS, LPFS or DPFS than those with microscopically positive (R1) or complete gross but microscopically unclear (Rx) resection margins. There was only one patient with gross residual disease (R2) so that further statistical analysis of the resection status is not reasonable. Also positive lymphonodal status or lymphangiosis carcinomatosa had no significant influence on the outcome in contrast to other studies [12, 39–41]. These findings may be explained by compensating effects of IOERT.

The cohort of patients with vulvar carcinoma was very small, so that it’s hard to make any statistical prediction, especially in a retrospective setting. In general, because of the rarity of recurrent vulvar cancer, only few clinical trials have been performed and the level of evidence for different treatment modalities is poor. In our cohort, survival seemed to be a little better compared to patients with recurrent cervical carcinoma, but not as good as survival of patients with recurrent endometrial carcinoma with a 5-year OS, LPFS and DPFS of 16.7, 20 and 30 %, respectively.

Treatment of recurrent gynecologic cancer remains a challenging issue. In general, pelvic exenteration can be considered in cases of recurrent or advanced-stage tumors with bladder and/or rectum infiltration neither extended to the pelvic side walls nor showing any distant metastases. With the possibility to use IOERT even patients with a recurrence of the pelvic side wall have a chance for good local control. But complications are likely, both of pelvic exenteration and IOERT, which have to be considered. The overall morbidity of pelvic exenteration is described in literature with about 70 % [12, 22, 23]. Apart from common early surgical complications such as large volume blood loss, sepsis or thromboembolic accidents, there may occur problems related to the denuded pelvic wall and floor like abscesses, intestinal fistulas and bowel obstructions. But there are also many long-term toxicities that must be taken into consideration, such as chronic and recurrent urinary infections, obstruction, pyelonephritis, renal insufficiency, loss of sexual function, pouch stones, etc. [12]. Most common complications of IOERT are peripheral nervous injury (18–30 %), gastrointestinal toxicity (15–24 %), and ureteral obstructions (3 %) [36, 42]. In our collective the postoperative mortality was higher than described in the literature with 8.3 % of patients dying from hemorrhagic shock. This might be due to the extent of resection including vessel reconstruction, prior radiotherapy, or even IOERT. The incidence of complications caused by IOERT described in literature [36, 42] is quite high. With the use of modern techniques as described above complications can be reduced. In general, it is hard to distinguish between complications caused by resection and those caused by IOERT retrospectively. 3 patients developed problems with the femoral head, in only one case an osteoradionecrosis could definitely be diagnosed, which was probably caused by prior pelvic EBRT. Most complications were healing disorders, lymphatic disorders and nerval affections that could be caused by both radiation treatment and/or resection. One of the patients with thrombosis had a vascular interponate.

All in all, patients with recurrent endometrial cancer seem to have the greatest benefit from resection combined with IOERT with 5-year survival rates of 50 %. In these patients, resection combined with IOERT can be regarded as valid curative treatment option and should be discussed with the patient. Patients with cervical or vulvar cancer have a much worse survival. With 5-year survival rates of 6.4 and 16.7 %, respectively, and even poor local control rates, this treatment should be considered a rather palliative one.

It is a very individual decision in which case of recurrent cervical carcinoma an extensive resection combined with IOERT is reasonable and ethically acceptable, especially in regard to the above-mentioned side effects. Patients should be selected carefully to define those who may benefit from that aggressive kind of treatment. Today there are much better diagnostic options considering technical development of MRI or PET-CT to identify patients with limited local recurrences eligible for resection and IOERT. The risk of distant progression in this patient collective should not be underestimated. In our collective, 55.6 % of all patients with recurrent cervical carcinoma developed distant metastases. In these cases or if the patient’s performance status isn’t good enough and the expected morbidity is high, other therapy options should be considered as well. Literature suggests that new targeted therapies show satisfactory results in advanced stages of recurrent cervical cancer. Tewari et al. showed that bevacizumab combined with chemotherapy resulted in a median survival of 17 months compared to 13.3 months with chemotherapy alone in the GOG 40 trial [43]. Toxicities were adequate and life quality not significantly affected by this therapy. In any case the radical surgical approach should be weighted carefully against other less invasive therapy options like the bevacizumab regime. For some patients even a combination of both modalities might be considered. On the other hand, advances in highly conformal radiotherapy techniques might offer new treatment options in combination with systemic approaches, thus avoiding extensive surgical approaches.

## Conclusion

The radical procedure of resection combined with IOERT seems to be a valid curative treatment option for patients with recurrent endometrial carcinoma with 5-year survival rates of 50 %. For patients with cervical or vulvar cancer this treatment should be considered a rather palliative one. Radical surgery should be weighted carefully against other treatment options like chemotherapy, targeted therapies or new highly conformal radiotherapy techniques.

### Ethics approval and consent to participate

Our research was carried out in compliance with the Declaration of Helsinki.

All data was collected retrospectively and in accordance with institutional ethical policies. Any patient information underlay professional discretion and data protection and was certainly treated confidentially. Due to the retrospective nature of any data collected and the fact that no additional examinations were carried out, no patients’ consent was obtained (in terms of self-research according to paragraph 15(3) LDSG BW).

### Consent for publication

Not applicable

## Additional file

Additional file 1: Figure S1. Kaplan-Meier curves of OS [A], LPFS [B] and DPFS [C] by different clinical parameters like histology, time to recurrence, lymphonodal status, lymphangiosis carcinomatosa, organinfiltration, resection status, and applied dose of IOERT. Further statistical analysis of possible predictive factors was performed. Histology, time to recurrence, lymphonodal status, lymphangiosis carcinomatosa, organinfiltration, resection status, and applied dose of IOERT were assessed. None of the mentioned parameters showed a statistically significant influence on OS, LPFS or DPFS. (PDF 357 kb)

## Abbreviations

CHT: chemotherapy
DPFS: distant progression-free survival
EBRT: external beam radiation therapy
Gy: gray
IOERT: intraoperative electron radiation therapy
LPFS: local progression-free survival
MeV: mega electron volt
MRI: magnetic resonance imaging
n.a.: not available
OS: overall survival
PET-CT: positron emission tomography – computed tomography
PFS: progression-free survival
