# Supplementary material for: Outcome of patients with local recurrent gynecologic malignancies after resection combined with intraoperative electron radiation therapy (IOERT)
Source: Radiat Oncol. 2016 Mar 18;11:44. doi: 10.1186/s13014-016-0622-x (PMC4797348; doi:10.1186/s13014-016-0622-x)
Supplement: Additional file 1: Figure S1. — Kaplan-Meier curves of OS [A], LPFS [B] and DPFS [C] by different clinical parameters like histology, time to recurrence, lymphonodal status, lymphangiosis carcinomatosa, organinfiltration, resection status, and applied dose of IOERT. Further statistical analysis of possible predictive factors was performed. Histology, time to recurrence, lymphonodal status, lymphangiosis carcinomatosa, organinfiltration, resection status, and applied dose of IOERT were assessed. None of the mentioned parameters showed a statistically significant influence on OS, LPFS or DPFS. (PDF 357 kb) [file 13014_2016_622_MOESM1_ESM.pdf]

Figure 2 Kaplan-Meier curves of OS [A], LPFS [B] and DPFS [C] by different clinical parameters like histology, time to recurrence, lymphonodal status, lymphangiosis carcinomatosa, organinfiltration, resection status and applicated dose of IOERT.

A

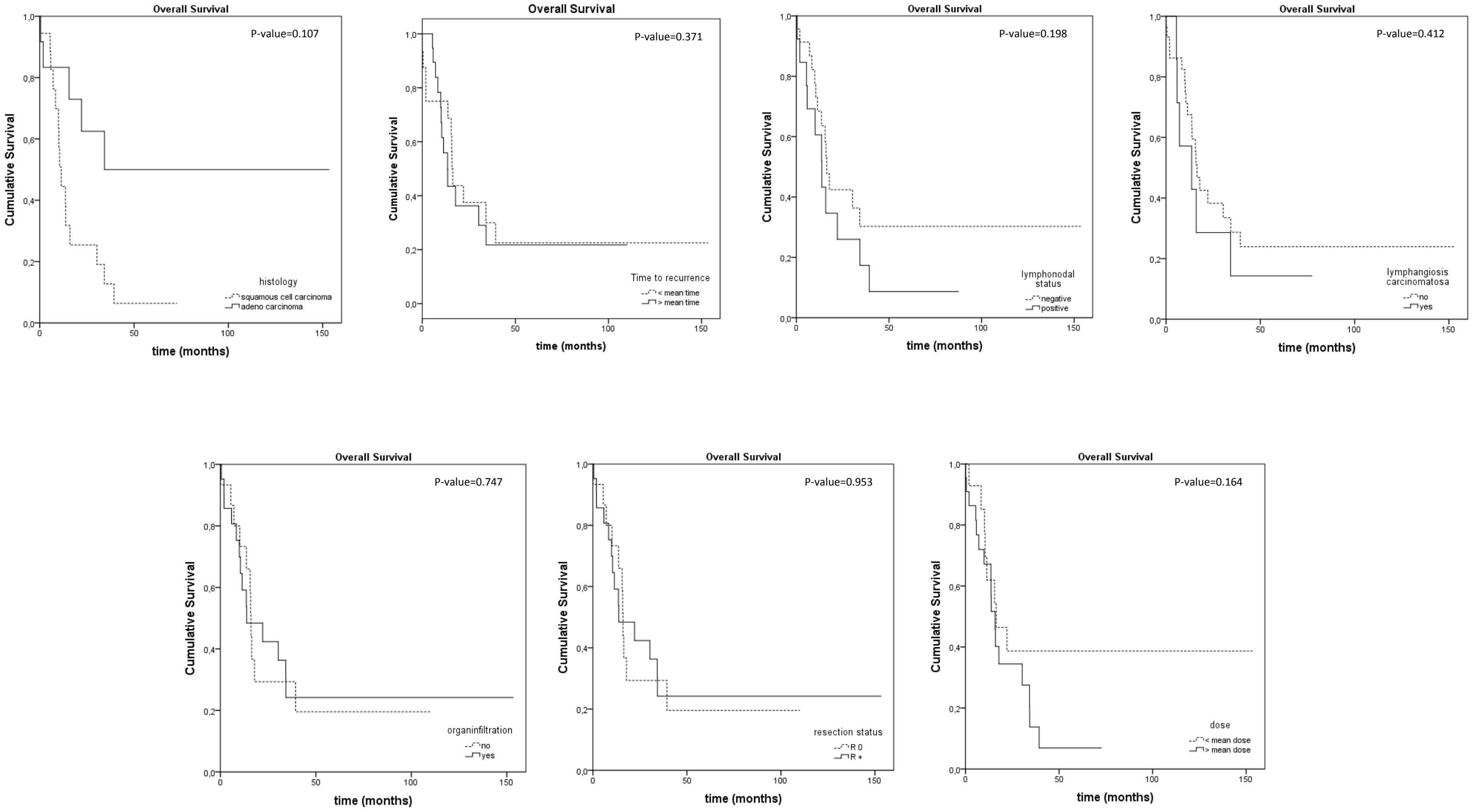

B

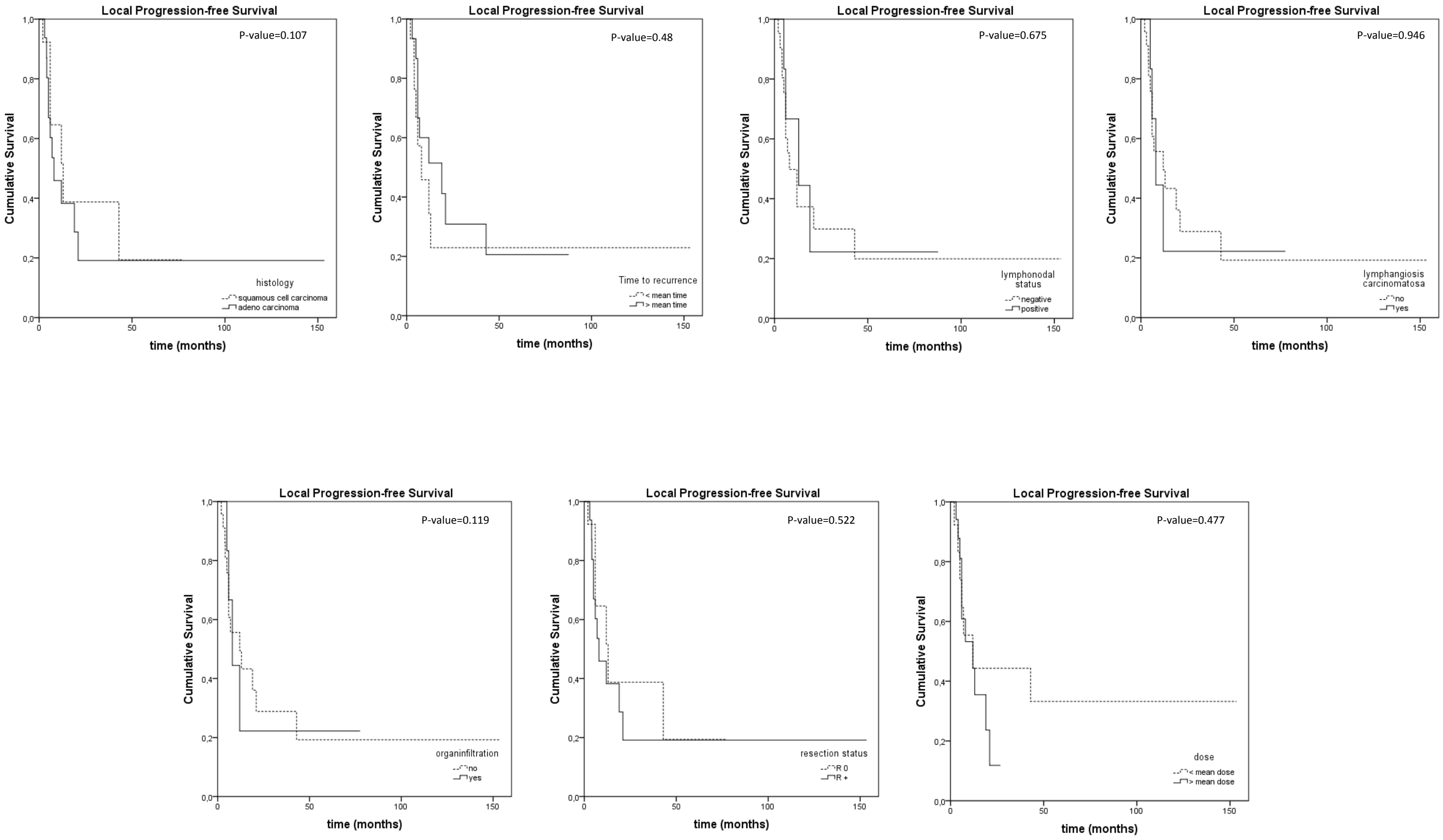

Local Progression-free Survival
P-value=0.107
Cumulative Survival
time (months)
histology
squamous cell carcinoma
adeno carcinoma
Local Progression-free Survival
P-value=0.48
Cumulative Survival
time (months)
Time to recurrence
< mean time
> mean time
Local Progression-free Survival
P-value=0.675
Cumulative Survival
time (months)
lymphonodal status
negative
positive
Local Progression-free Survival
P-value=0.946
Cumulative Survival
time (months)
lymphangiosis carcinomatosa
no
yes
Local Progression-free Survival
P-value=0.119
Cumulative Survival
time (months)
organinfiltration
no
yes
Local Progression-free Survival
P-value=0.522
Cumulative Survival
time (months)
resection status
R 0
R +
Local Progression-free Survival
P-value=0.477
Cumulative Survival
time (months)
dose
< mean dose
> mean dose

C

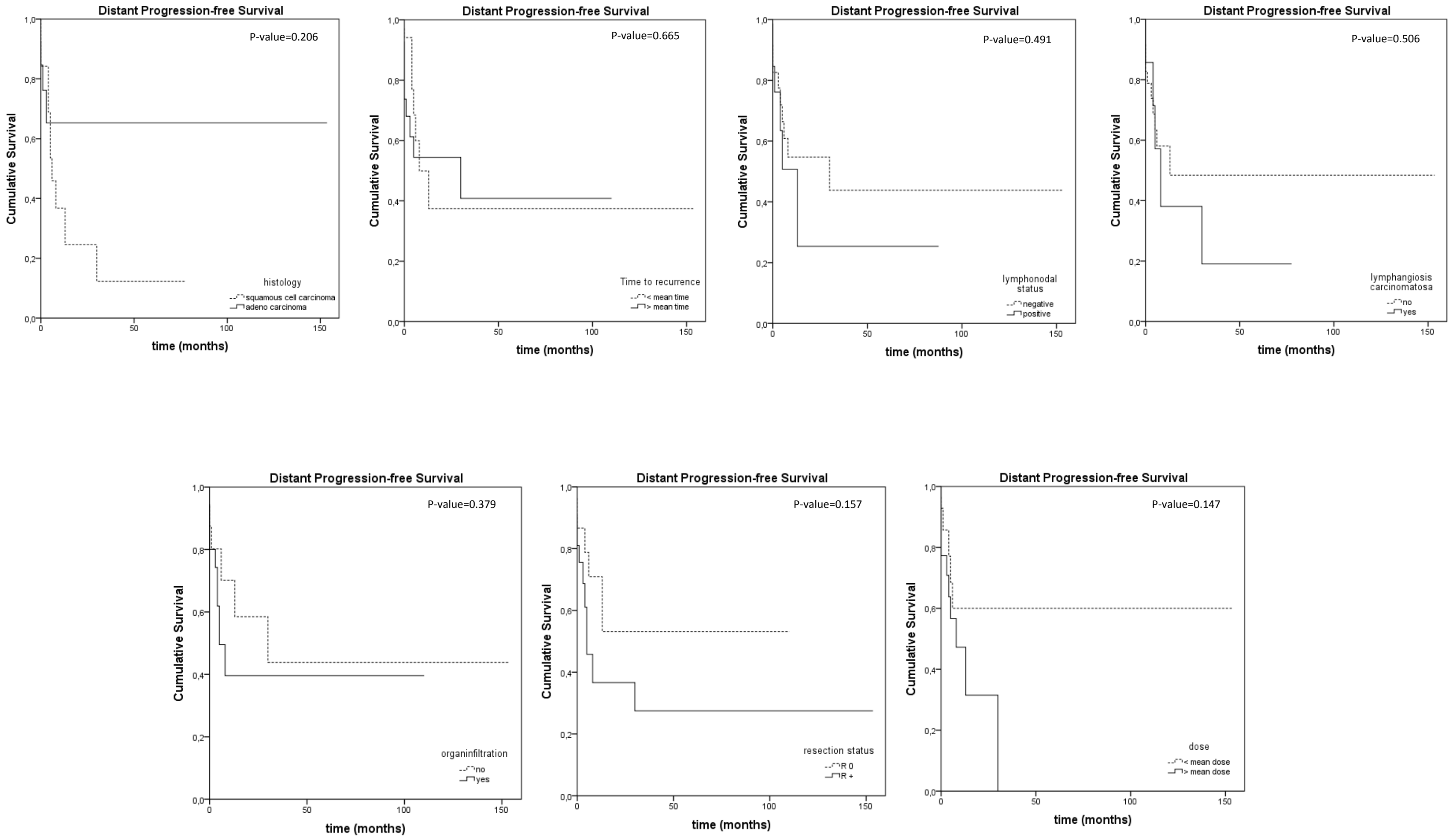
Distant Progression-free Survival
P-value=0.206
histology
squamous cell carcinoma
adeno carcinoma
Distant Progression-free Survival
P-value=0.665
Time to recurrence
< mean time
> mean time
Distant Progression-free Survival
P-value=0.491
lymphonodal status
negative
positive
Distant Progression-free Survival
P-value=0.506
lymphangiosis carcinomatosa
no
yes
Distant Progression-free Survival
P-value=0.379
organinfiltration
no
yes
Distant Progression-free Survival
P-value=0.157
resection status
R 0
R +
Distant Progression-free Survival
P-value=0.147
dose
< mean dose
> mean dose
Cumulative Survival
time (months)
